# Knowledge, attitudes and behaviours related to dietary salt among adults in the state of Victoria, Australia 2015

**DOI:** 10.1186/s12889-017-4451-0

**Published:** 2017-05-30

**Authors:** Carley A. Grimes, Sarah-Jane Kelley, Sonya Stanley, Bruce Bolam, Jacqui Webster, Durreajam Khokhar, Caryl A. Nowson

**Affiliations:** 1 Deakin University, Geelong, Australia, Institute for Physical Activity and Nutrition Research, Locked Bag 20000, Waurn Ponds, Geelong, VIC 3220 Australia; 2 The Victorian Health Promotion Foundation, VicHealth, 15-31 Pelham St, Carlton, VIC 3053 Australia; 3 The National Heart Foundation of Australia, Level 12 500 Collins St, Melbourne, VIC 3000 Australia; 4 The George Institute for Global Health, The University of Sydney, PO Box M201 Missenden Rd Camperdown, Sydney, NSW 2050 Australia

**Keywords:** Dietary salt, Dietary sodium, Knowledge, Attitude, Behaviour, Consumer, Australia

## Abstract

**Background:**

Information on consumer’s knowledge, attitudes and behaviours (KABs) related to salt can be used to inform awareness and education campaigns and serve as a baseline measure to monitor changes in KABs over time. The aim of this study was to determine KABs related to salt intake among Victorian adults.

**Methods:**

Cross-sectional survey conducted in Victorian adults aged 18–65 years. Participants were recruited from shopping centres located in Melbourne and Geelong and via online methods (Facebook and Consumer Research Panel) to complete an online survey assessing KABs related to dietary salt. Descriptive statistics (mean (SD) or n (%)) were used to report survey findings.

**Results:**

A total of 2398 participants provided a valid survey (mean age 43 years (SD 13), 57% female). The majority (80%) were born in Australia and 63% were the main household grocery shopper. The majority (89%) were aware of the health risks associated with a high salt intake. Eighty three percent believed that Australians eat too much salt. Three quarters (75%) correctly identified salt from processed foods as being the main source of salt in the diet. Less than a third (29%) of participants believed their own individual salt intake exceeded dietary recommendations and only 28% could correctly identify the maximum recommended daily intake for salt. Just under half (46%) of participants were concerned about the amount of salt in food. Almost two thirds (61%) of participants believed that there should be laws which limit the amount of salt added to manufactured foods and 58% agreed that it was difficult to find lower salt options when eating out.

**Conclusions:**

The findings of this study serve as a baseline assessment of KABs related to salt intake in Victorian adults and can be used to assess changes in salt related KABs over time. Public concern about salt is low as many people remain unaware of their own salt intake. An increased awareness of the excessive amount of salt consumed and increased availability of lower salt foods are likely to reduce population salt intake.

**Electronic supplementary material:**

The online version of this article (doi:10.1186/s12889-017-4451-0) contains supplementary material, which is available to authorized users.

## Background

The World Health Organization recommends a dietary salt intake of no more than 5 g/day for healthy adults [1]. However globally most people consume much higher amounts of salt, well beyond the recommended level [2]. Previous studies estimate the average dietary salt intake for Australian adults is between 8 and 9 g/day, almost twice the recommended daily intake [3–5]. This is similar to other industrialised countries, including the USA and UK, where salt consumption is approximately 10 g/day in males and 8 g/day in females [6, 7]. Approximately 75% of salt consumed in Western societies comes from processed foods and meals prepared in the food service industry, a much smaller proportion (15%) comes from salt added at the table and during cooking [8].

Sodium is an essential nutrient and for the body to function an intake of 10–20 mmol/d of sodium (salt equivalent 0.6–1.2 g/d) is required [8]. Excess salt intake is associated with the age-related rise in blood pressure [9]. In 2011–12 a third (32%) of Australian adults had hypertension [10], which represents a significant cause of premature death and disability in Australia [11]. Meta-analysis of randomised controlled trials have shown that among people with hypertension a 4.4 g/d reduction in salt intake (from a usual intake level of 9.5 g/d) significantly lowers systolic blood pressure by 5.4 mmHg millimetres of mercury (mm Hg) and diastolic blood pressure by 2.8 mmHg [12]. Among normotensive people a 4.4 g/d reduction (from a usual intake of 8.9 g/d) significantly lowers systolic and diastolic blood pressure by 2.4 mmHg and 1.0 mmHg, respectively [12]. Importantly, small shifts in the distribution of population blood pressure could provide considerable cardiovascular health gains [13, 14] and a 6 g/d reduction in salt intake would reduce stroke by 24% and coronary heart disease by 18% [14]. In 2013, Australia joined the WHO Member States in a global commitment to reduce population salt intake by 30% by the year 2025 [15].

Population salt reduction programs are usually multi-faceted, combining programs to change consumer behaviour with actions to get the food industry to reduce salt in foods [16]. The Victorian Health Promotion Foundation (VicHealth) is an independent statutory organisation funded by the State Government of Victoria. In 2015, VicHealth launched the multisector partnership to reduce population salt intake in Victoria through a combination of social marketing, industry engagement and research [17, 18]. This study was conducted to provide a baseline assessment of factors which influence salt intake in the Victorian population, to inform the planning, design and implementation of proposed salt reduction initiatives in Victoria. Specifically, the primary aim was to determine knowledge, attitudes and behaviours (KABs) related to salt intake among a sample of Victorian adults aged 18–65 years. In addition, we assessed the differences in salt related KABs by socio-demographic characteristics (i.e. sex, age group and socioeconomic status).

## Methods

### Study design and participants

This was a cross-sectional survey of Victorian adults aged 18–65 years. Participants were recruited using three strategies: i) shopping centre intercept survey; ii) online recruitment via Facebook; iii) online recruitment via a commercial research panel. Quotas were set for recruitment based on age and gender groups that reflected the population of Victoria [19]. Following the completion of the shopping centre and Facebook surveys it was determined that females and older participants (age groups 45–54 years and 55–65 years) were over-represented and hence to meet quotas a greater number of males and younger participants (25–34 years and 35–44 years) were targeted for recruitment via the online consumer research panel. Participants over the age of 65 years were excluded from the study, on the background that future salt related public awareness initiatives would primarily target those aged under 65 years. Participants completed an online survey assessing basic demographic characteristics and knowledge, attitudes and behaviours related to dietary salt intake. All participants provided informed consent and ethics approval was obtained by the Deakin University Human Ethics Advisory Group (Project No: HEAG-H 83_2015).

### Shopping centre intercept survey

Participants were recruited from shopping centres located in Greater Melbourne (3 sites) and Geelong (1 site) during September and November 2015. A total of 57 shopping centres were identified in the Greater Melbourne area and 8 in Geelong. The 2011 Socio-Economic Indexes for Areas (SEIFA) was used to match the postcode of each shopping centre with the corresponding Victorian SEIFA score based on the “Index of Relative Socio-Economic Advantage and Disadvantage” [20]. Following this, shopping centres were grouped into tertiles based on the assigned SEIFA score, for each region. To enable a spread of participants across different socio-economic stratum one shopping centre site was recruited from the bottom and the top tertile in Greater Melbourne; and one site from the bottom tertile in Geelong. During the project a fourth site was added to increase participation rates. The site selected was in the top tertile in Greater Melbourne as experience had proved higher participant numbers in this demographic profile. The final selection of shopping centres within each SEIFA tertile was dependent on stall costs and availability and obtaining permission to recruit shoppers obtained from the Centre management.

Research staff set up a stall within each site and invited passing-by shoppers to participate in the study. Adults aged greater than 65 years were excluded from participation (*n* = 156). Participants independently completed the online survey using tablets available on site. Data was primarily collected during the hours of 9:00 am to 5:00 pm Monday to Saturday, from September–October 2015. However, to capture a broad representation of adults, recruitment also occurred on Sunday’s and during late night shopping hours (Thursday evenings) at selected sites.

### Online survey (Facebook)

A ‘clicks to website’ advert was run on Facebook for 8 weeks during September to November 2015, inviting users to complete the online survey. Interested users clicked on the advert which diverted them to the plain language statement and consent form. After providing consent the participant was directed to the online survey. Parameters were set for the advert to be displayed to users aged 18–64 years residing in Victoria.

### Online survey (consumer research panel)

Participants were recruited through a commercial online research panel provider (Lightspeed GMI). The GMI research database is a database of individuals who have voluntarily registered themselves with GMI and are contacted periodically by GMI to take part in a variety of online surveys in return for reward points which they can redeem for monetary payments. After providing consent the participant was directed to the online survey. Data collection for this component of the project occurred during November, 2015.

### Survey instrument

A questionnaire containing 29 questions was developed to assess demographic characteristics and KAB related to dietary salt intake. Demographic characteristics assessed included age, sex, country of birth, language spoken at home, residential postcode and education level. Socioeconomic status (SES) was defined by educational attainment: i) low SES: includes those with some or no level of high school education ii) mid SES: includes those with a technical/trade Certificate or Diploma and iii) high SES: includes those with a university/tertiary qualification. Participants also reported on cardiovascular related co-morbidities, use of antihypertensive medication, household responsibility for grocery shopping, body weight and height. Body mass index (BMI) was calculated and participants were grouped into weight categories according to World Health Organization criteria [21]. The KAB questions were modelled on those used in previous salt related surveys [22–31]. Pilot testing with 20 adults of varying demographic background (age, gender and education status) resulted in minor revisions to improve readability and reduce the time required to complete the survey to approximately 10 min.

#### Knowledge

Six questions were used to assess participant’s knowledge related to dietary salt [see Additional file 1 Areas assessed included knowledge of the relationship between salt and sodium, dietary recommendations for salt intake, how population intake compares to recommendations, dietary sources of salt and the link between high salt intake and health outcomes. A range of categorical responses was provided for each question.

#### Attitudes

Four questions assessed attitudes. One question related to how the participant viewed their own intake of salt compared to recommendations. One block question used a 5-point Likert scale ranging from ‘strongly disagree’ to ‘strongly agree’ to assess a number of salt related attitudes, e.g. ‘my health would improve if I reduced the amount of salt in my diet’, ‘I believe salt needs to be added to food to make it tasty’ [Additional file 1]. For analyses, ‘disagree’/‘strongly disagree’ and ‘agree’/‘strongly agree’ were combined. Another block question assessed concern for a range of food related issues (e.g. healthy eating, sugar, fat and salt in diet) with responses on a scale of not at all concerned to extremely concerned. Participants were also asked who they believed was responsible for reducing population salt intake (e.g. government, food manufacturers, yourself) with responses including ‘not at all responsible’, ‘somewhat responsible’, ‘responsible’, ‘very responsible’ or ‘don’t know’. For analyses the responses of ‘responsible’ or ‘very responsible’ were combined.

#### Behaviours

Five questions assessed salt related behaviours, this included information on salt use during cooking and at the table and if a salt shaker is placed on the meal table during meal times. Responses included ‘always’, ‘often’, ‘sometimes’, ‘rarely’ or ‘never’. For analyses responses of ‘always’/‘often’ and ‘rarely’/‘never’ were combined. Participants were asked if they were trying to cut down on the amount of salt they eat (responses: ‘yes’, ‘no’, ‘don’t know’). A block question assessed a number of behaviours that participants may have engaged in within the previous month to reduce dietary salt, to which participants could respond ‘never do this’, ‘rarely do this’, ‘sometimes do this’, ‘often do this’, ‘always do this’ or ‘does not apply to me’. For analyses ‘never do this’/‘rarely do this’ and ‘often do this’/‘always do this’ were combined.

### Data analysis

The survey software instrument Qualtrics was used to deliver the surveys. All data were collated and analysed using the statistical program Stata/SE 14.0 (StataCorp LP). Descriptive statistics, mean and (standard deviation or standard error on weighted estimates) or n and (proportion %) were used to describe participant characteristics and responses to each of the survey questions. As the sample was over-representative of females and under-representative of younger participants, we created post-stratification weights, which weighted for sex and age (age groups: 18–24 y, 25–34 y, 35–44 y, 45–54 y, 55–65 y) consistent with the population of Victoria [19]. For analyses which related to the whole sample post-stratification weights were applied using the probability weight (pweight) specification in Stata/SE. To assess differences in frequencies of categorical responses by sociodemographic sub-groups (i.e. sex, age-group and SES) chi-square tests were used. A *p*-value of <0.05 was considered statistically significant.

## Results

A total of 2559 participants agreed to complete the online survey. The response rate for the shopping centre intercept survey and online consumer research panel was 19.4% and 13.7%, respectively. It was not possible to determine the response rate for participants recruited via Facebook. Participants who did not answer any of the survey questions (*n* = 46, 1.8%) or did not complete the survey to the end were excluded from the analysis (*n* = 115, 4.5%). The final analytical sample was 2398 (93.7%). Of these participants, 1621 (67.6%) completed the survey through the online consumer research panel, 404 (16.8%) completed the survey via Facebook and 373 (15.6%) completed the survey at the shopping centres.

Just over half (56%) of the sample were female and the majority (80%) were born in Australia (Table 1). The average age of both males and females was 43 years and there was a relatively even distribution of participants across age groups. Approximately half of participants were from a high socio-economic background and 40% were in the healthy weight range category. In comparison to the Victorian population, people from a higher socioeconomic background, females and elderly aged 55–65 years were overrepresented, while younger people aged between 18 and 24 years were underrepresented (Table 1). The demographic characteristics of participants differed by recruitment method and these are shown in Additional file 2: Table S1.

**Table 1:** Demographic characteristics of participants (*n* = 2398)

Characteristic	Survey sample (unweighted)		Survey sample (weighted)^a^	Victorian Population
	n or mean	% or SD	% or mean	%
Gender				
Male	1046	43.6%	47.5%	49.3%^c^
Female	1352	56.4%	52.5%	51.7%
Age (years) (mean, SD)	42.7	13.4	41.3	
Males (mean, SD)	43.0	12.9	40.7	
Females (mean, SD)	42.6	13.8	42.0	
Age group				
18–24 y	251	10.5%	14.5%	15.0%^c^
25–34 y	512	21.3%	21.4%	22.1%
35–44 y	527	22.0%	21.8%	22.5%
45–54 y	514	21.4%	20.4%	21.1%
55–65 y	594	24.8%	22.0%	19.3%
Country of Birth				
Australia	1915	79.9%	80.0%	
United Kingdom	86	3.5%	3.5%	
New Zealand	29	1.2%	1.2%	
Italy	10	0.4%	0.4%	
Greece	11	0.5%	0.5%	
China	30	1.2%	1.3%	
Vietnam	14	0.6%	0.6%	
Lebanon	4	0.2%	0.2%	
Other	271	11.3%	11.1%	
Prefer not to answer or don’t know	28	1.2%	1.2%	
Do you speak a language other than English at home?				
Yes	409	17.0%	17.8%	
No, English only	1969	82.1%	81.3%	
Prefer not to answer or don’t know	20	0.8%	0.9%	
Socioeconomic status^b^				
High SES	1020	42.9%	43.0%	28.1%^d^
Mid SES	675	28.4%	27.7%	27.0%
Low SES	682	28.7%	29.3%	42.9%
Height (cm) (mean, SD)	169.3	10.2	169.8	
Weight (Kg) (mean, SD)	77.6	18.7	77.5	
BMI (kg/m^2^) (mean, SD)	27	6.1	26.8	
Weight category				
Underweight	68	3.2%	3.5%	
Healthy weight	846	39.6%	40.7%	
Overweight	690	32.3%	31.9%	
Obese	532	24.9%	23.9%	

^a^Demographic characteristics weighted for age and gender

^b^ *n* = 2377 as participants who responded “don’t know” *n* = 3 or “prefer not to answer” *n* = 18 were excluded.

^c^Data taken from the 2011 Australian Census and reflects the proportion of adults aged 18–65 years residing in Victoria [19]

^d^Data taken from 2016 Survey of Education and Work and includes information on educational attainment in Victorian adults aged 15–74 years. Consistent with our definition of SES we grouped the following responses into each group. Low SES: ‘Year 12 or equivalent’, ‘Year 11’, ‘Year 10’ or ‘Below Year 10’; mid SES: ‘Certificate III/IV’ or ‘Advanced Diploma/Diploma’; high SES: ‘Bachelor Degree’, ‘Graduate Diploma/Graduate Certificate’ or ‘Postgraduate Degree’ [52]

Just under a third of the sample (29.4%) reported they had previously been diagnosed or suffered from a chronic condition, with the most common being high blood pressure (21.4%). National estimates for high blood pressure, based on a measured blood pressure reading of SBP ≥140 mmHg and/or DBP ≥90 mmHg or reported use of hypertensive medications, among Australian adults is 31.6% [32]. Of those with high blood pressure (*n* = 514), 74.9% reported taking medication to control their blood pressure. Two thirds (69.0%) of participants reported that they were the primary person responsible for household grocery shopping, whilst 21.0% shared the responsibility.

### Knowledge and attitudes related to salt intake

**Fig. 1:**
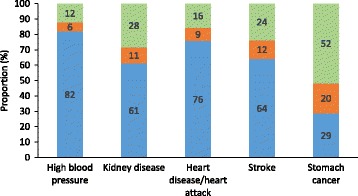
Knowledge of conditions linked with a high salt intake (*n* = 2398)^1^. Legend:  Yes,  No,  Don’t know. ^1^Estimates weighted for age and gender

The majority of participants (90%) knew that eating too much salt could damage their health (Table 2). Most were aware of the relationship between high salt intake and high blood pressure (83%) and heart disease/heart attack (77%). Approximately two thirds knew of the relationship between salt intake and stroke and kidney disease, however far fewer were aware of links with stomach cancer (Fig. 1). Only a third (33%) of participants could correctly identify the relationship between salt and sodium. Three quarters (75%) knew that most salt in the Australian diet comes from processed foods. Most (83%) participants believed Australians eat either far too much or too much salt but only 28% could correctly identify the recommended maximum amount of salt to eat per day and less than a third (29%) of participants believed their own individual salt intake would exceed dietary guidelines (Table 2).

**Table 2:** Knowledge and attitudes related to dietary salt (*n* = 2398)^a^

Question	Weighted %	SE
Do you think that eating too much salt could damage your health?		
**Yes**	**89.8**	0.7
No	4.5	0.5
Don’t know	5.7	0.5
On Australian food products, information about the amount of sodium within a food product is displayed on the food label. What is the relationship between salt and sodium?		
They are exactly the same	45.6	1.0
**Salt contains sodium**	**33.1**	1.0
Sodium contains salt	3.4	0.4
Don’t know	17.9	0.8
Which of the following do you think is the main source of salt in the Australian diet?		
Salt added during cooking or at the table	17.1	0.8
**Salt from processed foods such as breads, sausages and cheese**	**74.8**	0.9
Salt from natural food sources	2.5	0.3
Don’t know	5.5	0.5
In general, how much salt do you think Australians eat?		
**Far too much**	**33.1**	1.0
**Too much**	**50.1**	1.0
Just the right amount	7.9	0.6
Too little	1.5	0.3
Far too little	0.3	0.1
Don’t know	7.2	0.5
Health professionals recommend that we should eat no more than a certain amount of salt each day. How much salt do you think this is?		
3 g (about 1/2 a teaspoon)	27.1	0.9
**5 g (about 1 teaspoon)**	**27.8**	0.9
8 g (about 1 and a 1/2 teaspoons)	10.4	0.7
10 g (about 2 teaspoons)	5.0	0.5
15 g (about 3 teaspoons)	1.4	0.3
Don’t know	28.3	0.9
How do you think your daily salt intake compares to the amount of salt recommended by health professionals?		
I eat less salt than recommended	18.3	0.8
I eat about the right amount of salt	36.4	1.0
I eat more salt than recommended	29.0	1.0
I don’t know	16.3	0.8

^a^Correct responses for knowledge questions are in bold

Overall there were differences in salt related knowledge across socio-demographic sub-groups (Table 3). Generally females were more knowledgeable, with a greater proportion aware of high salt intakes within the population, along with the main dietary source of salt and health outcomes related with excess intake (Table 3). Conversely, more males were able to correctly identify the relationship between salt and sodium. With respects to age, younger participants were more likely to understand the salt and sodium relationship and the recommended amount of salt to consume. Whereas, older participants were more likely to know the main dietary source of salt and that Australians consume too much. There was no association between age group and knowledge that excess salt can damage health, however older participants were more likely to be aware of the link with certain specific health conditions (e.g. high blood pressure, heart disease and stroke). Not all knowledge items differed across socioeconomic groups, however participants of higher socioeconomic background were more likely to correctly respond to some items (e.g. salt and sodium relationship, main dietary source of salt, excess salt linked to worse health) (Table 3).

**Table 3:** Knowledge and attitudes related to dietary salt by demographic characteristics (*n* = 2398)^a, b^

Knowledge related questions	Sex (*n* = 2398)			Age group (years) (*n* = 2398)						SES (*n* = 2377)			
	Male	Female	*P*-value	18–24	25–34	35–44	45–54	55–65	*P*-value	Low	Mid	High	*P*-value
	%	%		%	%	%	%	%		%	%	%	
On Australian food products information about the amount of sodium within a food product is displayed on the food label. What is the relationship between salt and sodium?													
*Correct answer - Salt contains sodium*	*35.7*	*30.3*	***0.006***	*44.2*	*34.6*	*29.8*	*29.8*	*31.1*	***<0.001***	*24.2*	*29.8*	*40.4*	***<0.001***
Incorrect answer	64.3	69.7		55.8	65.4	70.2	70.2	68.9		75.8	70.2	59.6	
In general, how much salt do you think Australians eat?													
*Correct answer - Far too much or Too much*	*79.3*	*87.3*	***<0.001***	*81.3*	*79.5*	*82.7*	*86.2*	*87.7*	***0.002***	*84.0*	*84.0*	*84.4*	*0.965*
Incorrect answer	20.7	12.7		18.7	20.5	17.3	13.8	12.3		16.0	16.0	15.6	
Which of the following do you think is the main source of salt in the Australian diet?													
*Correct answer - Salt from processed foods* *such as breads, sausages and cheese*	*67.3*	*82.5*	***<0.001***	*73.3*	*69.1*	*70.0*	*78.6*	*85.7*	***<0.001***	*72.7*	*76.7*	*78.3*	***0.027***
Incorrect answer	32.7	17.5		26.7	30.9	30.0	21.4	14.3		27.3	23.3	21.7	
Health professionals recommend that we should eat no more than a certain amount of salt each day. How much salt do you think this is?													
*Correct answer - 5 g (about 1 teaspoon)*	*25.5*	*29.1*	*0.054*	*33.9*	*29.7*	*28.5*	*24.7*	*24.6*	***0.025***	*25.2*	*27.3*	*29.6*	*0.134*
Incorrect answer	74.5	70.9		66.1	70.3	71.5	75.3	75.4		74.8	72.7	70.4	
Do you think that eating too much salt could damage your health?													
*Correct answer - Yes*	*86.4*	*93.2*	***<0.001***	*91.2*	*89.3*	*87.9*	*90.3*	*92.8*	*0.074*	*88.1*	*91.4*	*91.6*	***0.039***
Incorrect answer	13.6	6.8		8.8	10.7	12.1	9.7	7.2		11.9	8.6	8.4	
Which, if any, of the following conditions do you think is linked to eating too much salt?													
High blood pressure													
*Correct answer - Yes*	*80.5*	*84.5*	***0.011***	*78.5*	*77.3*	*81.6*	*84.4*	*88.7*	***<0.001***	*79.9*	*81.0*	*86.7*	***<0.001***
Incorrect answer	19.5	15.5		21.5	22.7	18.4	15.6	11.3		20.1	19.0	13.3	
Kidney disease													
*Correct answer - Yes*	*55.7*	*67.1*	***<0.001***	*59.8*	*59.2*	*62.6*	*60.7*	*66.5*	*0.099*	*53.2*	*61.8*	*68.8*	***<0.001***
Incorrect answer	44.3	32.9		40.2	40.8	37.4	39.3	33.5		46.8	38.2	31.2	
Heart disease/heart attack													
*Correct answer - Yes*	*70.8*	*81.4*	***<0.001***	*72.1*	*71.9*	*74.2*	*80.4*	*82.2*	***<0.001***	*77.1*	*76.4*	*77.4*	*0.901*
Incorrect answer	29.2	18.6		27.9	28.1	25.8	19.7	17.9		22.9	23.6	22.6	
Stroke													
*Correct answer - Yes*	*60.1*	*69.0*	***<0.001***	*58.2*	*58.0*	*62.6*	*66.3*	*75.4*	***<0.001***	*62.5*	*65.3*	*67.3*	*0.115*
Incorrect answer	39.9	31.0		41.8	42.0	37.4	33.7	24.6		37.5	34.7	32.7	
Stomach cancer													
*Correct answer - Yes*	*28.8*	*28.8*	*0.998*	*29.9*	*34.4*	*29.6*	*27.0*	*24.2*	***0.005***	*26.4*	*29.3*	*30.0*	*0.254*
Incorrect answer	71.2	71.1		70.1	65.6	70.4	73.0	75.8		73.6	70.7	70.0	
Attitude question													
How do you think your daily salt intake compares to the amount of salt recommended by health professionals?													
I eat less salt than recommended	16.9	19.9	0.135	13.2	16.2	15.4	20.4	24.2	<0.001	19.9	19.1	17.5	<0.001
I eat about the right amount of salt	36.6	37.3		31.9	35.7	36.8	34.8	42.3		31.4	40.2	39.3	
I eat more salt than recommended	28.8	27.7		41.0	32.4	29.0	27.1	19.4		27.7	25.0	30.8	
I don’t know	17.7	15.1		13.9	15.7	18.8	17.7	14.1		21.0	15.7	12.4	

^a^Correct responses for knowledge questions are shown in italics. ‘Don’t know’ responses were coded as incorrect

^b^Association between categorical variables assessed by Chi-square test. Significant findings (i.e. *P* < 0.05) are shown in bold

**Fig. 2:**
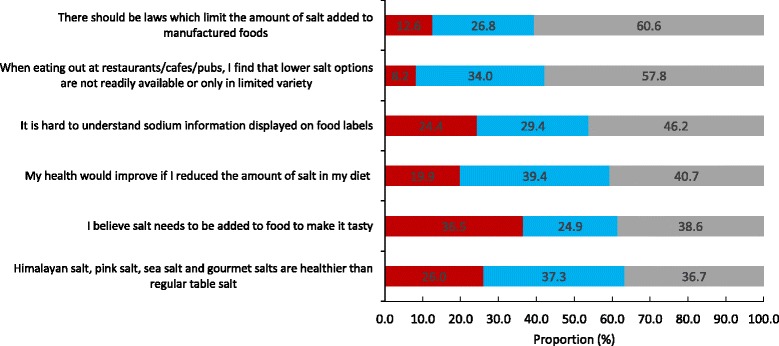
Level of agreement with attitude statements related to salt intake (*n* = 2398)^1^. Legend:  Disagree or Strongly Disagree,  Neither Agree nor Disagree,  Agree or Strongly Agree. ^1^ Estimates weighted for age and gender

Figure 2 shows participants level of agreement on a range of attitudes related to salt intake. Almost two thirds (61%) of participants agreed that there should be laws which limit the amount of salt added to manufactured foods. Females and older participants were more likely to agree with this statement, whereas there were no difference in agreement across socioeconomic groups (Table 4). Overall about half agreed that it was difficult to find low salt options when eating out (58%). Across sub-groups this was more commonly reported amongst females, older participants and those of higher socioeconomic background (Table 4). Forty six percent of participants reported that it was hard to understand sodium information displayed on food labels, with differences in sub-groups shown in Table 4. Overall less than half (41%) believed their health would improve if they reduced the amount of salt in their diet, however by sub-group more males, younger participants and those of higher socioeconomic background held this belief (Table 4). In total, about a third (39%) agreed that salt should be added to food to make it tasty, with more males and younger participants agreeing with this statement (Table 4). Similarly about a third (37%) agreed that speciality salts are healthier than regular table salt, with more females, younger participants and participants of higher socioeconomic background in agreement with this statement (Table 4). Of note, generally speaking a greater proportion of males, younger participants and those of lower socio-economic background neither agreed nor disagreed with attitude statements (Table 4).

**Table 4:** Level of agreement with attitude statements related to salt intake by demographic characteristics^a^

Attitude statement	Sex (*n* = 2398)			Age group (years) (*n* = 2398)						SES (*n* = 2377)			
	Male	Female	*P*-value	18–24	25–34	35–44	45–54	55–65	*P*-value	Low	Mid	High	*P*-value
	%	%		%	%	%	%	%		%	%	%	
There should be laws which limit the amount of salt added to manufactured foods													
Agree or strongly agree	56.0	65.4	**<0.001**	50.6	57.8	60.3	64.0	67.4	**<0.001**	57.8	60.6	64.6	0.055
Neither agree nor disagree	30.1	23.7		29.5	29.7	29.8	24.1	21.7		28.0	27.4	24.4	
Disagree or strongly disagree	13.9	10.9		19.9	12.5	9.9	11.9	10.9		14.2	12.0	11.0	
When eating out at restaurants/cafes/pubs, I find that lower salt options are not readily available or only in limited variety													
Agree or strongly agree	53.9	61.7	**<0.001**	54.2	57.0	56.6	57.2	63.6	**0.019**	52.2	56.0	64.5	**<0.001**
Neither agree nor disagree	38.0	30.5		36.3	32.2	35.5	36.0	30.7		38.9	36.0	28.4	
Disagree or strongly disagree	8.1	7.8		9.5	10.8	7.9	6.8	5.7		8.9	8.0	7.1	
It is hard to understand sodium information displayed on food labels													
Agree or strongly agree	45.8	47.5	**<0.001**	41.4	51.4	46.5	45.1	46.6	**0.023**	45.0	47.1	48.0	**0.019**
Neither agree nor disagree	33.8	24.8		32.7	26.5	32.5	28.4	25.8		32.4	29.2	25.2	
Disagree or strongly disagree	20.4	27.7		25.9	22.1	21.0	26.5	27.6		22.6	23.7	26.8	
My health would improve if I reduced the amount of salt in my diet													
Agree or strongly agree	43.7	37.6	**<0.001**	51.8	38.9	43.1	36.4	37.4	**<0.001**	37.2	34.1	46.5	**<0.001**
Neither agree nor disagree	42.1	37.8		27.1	42.2	37.0	42.2	42.9		43.0	44.9	33.7	
Disagree or strongly disagree	14.2	24.6		21.1	18.9	19.9	21.4	19.7		19.8	21.0	19.8	
I believe salt needs to be added to food to make it tasty													
Agree or strongly agree	43.2	33.9	**<0.001**	38.2	44.7	41.0	34.1	32.8	**<0.001**	36.9	34.9	41.0	**0.010**
Neither agree nor disagree	27.0	22.8		26.7	26.2	24.1	26.6	21.1		27.0	26.7	20.9	
Disagree or strongly disagree	29.8	43.3		35.1	29.1	34.9	39.3	46.1		36.1	38.4	38.1	
Himalayan salt, pink salt, sea salt and gourmet salts are healthier than regular table salt													
Agree or strongly agree	34.5	38.4	**<0.001**	40.2	43.9	39.3	33.7	29.3	**<0.001**	34.9	37.5	37.8	**<0.001**
Neither agree nor disagree	42.4	32.9		36.7	34.4	39.1	39.9	35.2		44.1	40.9	29.0	
Disagree or strongly disagree	23.1	28.7		23.1	21.7	21.6	26.4	35.5		21.0	21.6	33.2	

^a^Association between categorical variables assessed by Chi-square test. Significant findings (i.e. *P* < 0.05) are shown in bold

**Fig. 3:**
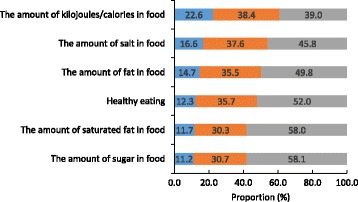
Level of public concern for food related issues (*n* = 2398)^1^. Legend:  Not at all or not very concerned,  Somewhat concerned,  Very or extrememly concerned. ^1^ Estimates weighted for age and gender

The level of public concern regarding food related issues was relatively high, with 39–58% of participants reporting that they were either very or extremely concerned with each food related issue (Fig. 3). Sugar and saturated fat were the nutrients of most concern, whereas just under half (46%) of participants were very or extremely concerned about the amount of salt in food. With the exception of the amount of kilojoules in food, across all other food related issues females, compared to males, were significantly more likely to report their concern for each issue (Table 5). Generally speaking across each age-group, the proportion of participants reporting concern for each food related issue increased (Table 5). With regards to socioeconomic differences those of higher socioeconomic were more likely to be concerned about all of the food related issues, with the exception of the amount of fat in food where there was no difference for concern across SES groups (Table 5).

**Table 5:** Level of public concern for food related issues by demographic characteristics^a^

Food related issue	Sex (*n* = 2398)			Age group (years) (*n* = 2398)						SES (*n* = 2377)			
	Male	Female	*P*-value	18–24	25–34	35–44	45–54	55–65	*P*-value	Low	Mid	High	*P*-value
	%	%		%	%	%	%	%		%	%	%	
The amount of sugar in food													
Very or extremely concerned	53.6	63.9	**<0.001**	43.8	55.3	58.6	58.0	71.6	**<0.001**	54.4	57.5	64.8	**<0.001**
Somewhat concerned	32.0	29.0		36.3	31.0	31.5	34.0	22.9		30.6	32.9	28.1	
Not at all or not very concerned	14.4	7.1		19.9	13.7	9.9	8.0	5.5		15.0	9.6	7.1	
The amount of saturated fat in food													
Very or extremely concerned	53.5	64.0	**<0.001**	47.0	54.9	56.6	59.0	71.4	**<0.001**	56.3	58.2	62.9	**0.013**
Somewhat concerned	31.9	28.1		35.5	30.9	32.8	31.7	22.0		30.4	31.9	27.9	
Not at all or not very concerned	14.6	7.9		17.5	14.2	10.6	9.3	6.6		13.3	9.9	9.2	
Healthy eating													
Very or extremely concerned	44.1	59.9	**<0.001**	48.6	55.1	47.3	51.4	59.6	**<0.001**	45.2	49.5	61.1	**<0.001**
Somewhat concerned	40.3	31.2		39.0	30.9	38.5	37.4	32.5		39.6	37.5	30.7	
Not at all or not very concerned	15.6	8.9		12.4	14.0	14.2	11.3	7.9		15.2	13.0	8.2	
The amount of fat in food													
Very or extremely concerned	46.4	54.3	**<0.001**	42.2	47.1	47.8	51.6	59.9	**<0.001**	50.2	49.8	52.7	0.366
Somewhat concerned	37.2	34.2		34.3	34.4	39.1	37.3	32.2		35.3	35.4	35.4	
Not at all or not very concerned	16.4	11.5		23.5	18.5	13.1	11.1	7.9		14.5	14.8	11.9	
The amount of salt in food													
Very or extremely concerned	41.8	51.1	**<0.001**	30.3	42.2	43.6	46.9	61.5	**<0.001**	45.4	45.5	49.7	**0.014**
Somewhat concerned	40.3	34.9		41.8	39.4	38.9	39.1	30.4		35.5	38.9	37.2	
Not at all or not very concerned	17.9	14.0		27.9	18.4	17.5	14.0	8.1		19.1	15.6	13.1	
The amount of kilojoules/cal in food													
Very or extremely concerned	38.1	40.5	0.058	32.3	43.2	37.9	35.4	44.3	**<0.001**	37.0	36.1	43.9	**0.001**
Somewhat concerned	37.8	39.4		35.8	32.2	41.0	41.8	40.7		37.8	40.9	37.7	
Not at all or not very concerned	24.1	20.1		31.9	24.6	21.1	22.8	15.0		25.2	23.0	18.4	

^a^Association between categorical variables assessed by Chi-square test. Significant findings (i.e. *P* < 0.05) are shown in bold

**Fig. 4:**
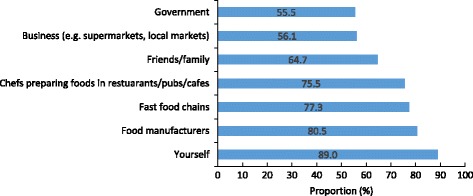
Proportion of people who believe the following groups are responsible for population salt reduction (*n* = 2398)^1^. ^1^Estimates weighted for age and gender

Participants reported that the responsibility for reducing the amount of salt consumed by the Australian population extends to individuals (89%), food manufacturers (81%), fast food chains (77%) and chefs (76%) (Fig. 4). For most of the identified groups (i.e. food manufacturers, friends/family and fast food chains), the different socioeconomic sub-groups agreed on their level of responsibility for salt reduction. Some differences observed between sub-groups, included females being more likely than males to believe salt reduction was the responsibility of the individual and younger participants and those of higher socioeconomic backgrounds more likely to indicate the government was responsible for action (Table 6).

**Table 6:** Proportion of people who believe the following groups are responsible for population salt reduction by demographic characteristics^a b^

Group	Sex			Age group (years)						SES			
	Male	Female	*P*-value	18–24	25–34	35–44	45–54	55–65	*P*-value	Low	Mid	High	*P*-value
	%	%		%	%	%	%	%		%	%	%	
Government (sex & age group *n* = 2278, SES *n* = 2262)													
Responsible	60.3	56.3	0.146	58.6	63.7	60.4	56.7	52.0	**0.011**	54.3	57.6	60.3	**0.009**
Somewhat responsible	26.1	28.2		27.2	24.4	27.2	27.8	29.4		27.0	27.7	27.5	
Not responsible	13.6	15.5		14.2	11.9	12.5	15.5	18.6		18.7	14.7	12.2	
Business (e.g. supermarkets, local markets) (sex & age group *n* = 2294, SES *n* = 2278)													
Responsible	61.3	55.9	**0.032**	59.8	62.8	58.7	55.8	55.1	0.182	53.8	59.1	60.1	**0.005**
Somewhat responsible	22.8	25.3		24.7	23.3	23.8	24.1	25.4		24.8	22.3	25.3	
Not responsible	15.9	18.8		15.5	13.9	17.5	20.1	19.5		21.4	18.6	14.6	
Friends/family (sex & age group *n* = 2311, SES *n* = 2295)													
Responsible	67.5	66.8	0.574	67.9	68.2	68.5	65.9	65.7	0.915	65.9	68.5	66.8	0.614
Somewhat responsible	23.2	22.6		22.9	22.1	22.0	24.7	22.8		23.0	21.4	23.9	
Not responsible	9.3	10.6		9.2	9.7	9.5	9.4	11.5		11.1	10.1	9.3	
Chefs preparing foods in restaurants/pubs/cafes(sex & age group *n* = 2324, SES *n* = 2307)													
Responsible	76.0	79.9	**0.003**	74.5	79.3	78.2	79.1	78.1	0.634	77.9	76.4	79.5	0.280
Somewhat responsible	17.8	16.7		21.0	16.5	17.8	16.7	16.0		16.4	19.3	16.4	
Not responsible	6.2	3.4		4.5	4.2	4.0	4.2	5.9		5.7	4.3	4.1	
Fast food chains (sex & age group *n* = 2301, SES *n* = 2284)													
Responsible	80.0	81.1	0.796	79.8	80.4	82.4	78.9	81.3	0.145	79.6	80.2	81.6	0.298
Somewhat responsible	11.6	11.0		13.0	13.3	10.6	11.3	9.1		10.7	12.7	10.7	
Not responsible	8.4	7.9		7.2	6.3	7.0	9.8	9.6		9.7	7.1	7.7	
Food manufacturers (sex & age group *n* = 2319, SES *n* = 2302)													
Responsible	81.8	84.7	0.139	82.3	85.1	83.5	83.3	82.5	0.063	81.3	84.3	84.3	0.529
Somewhat responsible	11.6	10.3		12.8	11.6	11.5	9.9	9.5		12.3	10.2	10.4	
Not responsible	6.6	5.0		4.9	3.3	5.0	6.8	8.0		6.4	5.5	5.3	
Yourself (sex & age group *n* = 2290, SES *n* = 2274)													
Very responsible or responsible	91.8	95.2	**0.004**	93.5	93.2	92.9	93.1	95.5	0.569	93.3	93.1	94.5	0.685
Somewhat responsible	6.9	3.9		4.8	6.0	5.5	5.9	3.8		5.3	5.8	4.7	
Not responsible	1.3	0.9		1.7	0.8	1.6	1.0	0.7		1.4	1.1	0.8	

^a^Association between categorical variables assessed by Chi-square test. Significant findings (i.e. *P* < 0.05) are shown in bold

^b^Participants who responded ‘don’t know’ were removed from this analysis. The total number of participants for each sub-group is indicated for each question Note the number of participants for the SES sub-group is lower as within the sample *n* = 21 participants did not provide information on educational attainment

### Behaviours related to salt intake

**Fig. 5:**
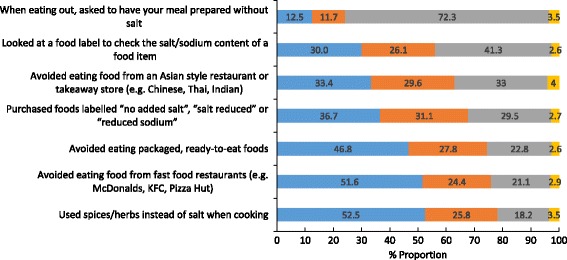
Behavioural practices to reduce salt intake performed in the past month (*n* = 2398)^1^. Legend:  Often or Always do this,  Sometimes do this,  Never or Rarely do this,  Does not apply to me. ^1^Estimates weighted for age and gender

**Fig. 6:**
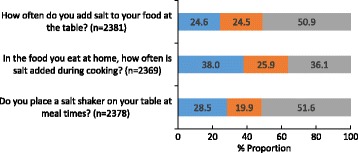
Discretionary salt use behaviours ^1,2^. Legend:  Always or Often,  Sometimes,  Rarely or Never. ^1^Participants who responded ‘don’t know’ were removed from analysis (*n* = 20 table salt, *n* = 29 cooking salt, *n* = 17 salt shaker on the table). ^2^Estimates weighted for age and gender

Forty percent of all participants reported that they were trying to cut down on the amount of salt they consume. There were no gender or socio-economic background differences in those reporting to cut down on salt, however compared to younger participants, older participants were more likely to report this behaviour (Table 7). Within the total sample, the most commonly reported behaviours to lower salt intake in the past month included using spices/herbs instead of salt when cooking, avoiding eating from fast food outlets and avoiding eating packaged foods, these behaviours were reported by about half of the sample (Fig. 5). Fewer participants, about a third, reported that they purchased salt reduced foods or used the sodium information on food labels. Figure 6 shows the proportion of participants who report using salt at the table and during cooking. With the exception of one behaviour (i.e. asking to have a meal your meal prepared without salt), females were more likely than males to report engaging in all other salt related behaviours (Table 8). A similar pattern existed for older participants, compared to younger participants and for those of higher socioeconomic background (Table 8). The reported use of cooking salt was higher than table salt (Fig. 5) and just over a quarter of participants reported that they always or often place a salt shaker on the table at meal times. Compared to females, males were more likely to report use of table salt, cooking salt and placing a salt shaker on the table (Table 9). Similarly, younger participants were more likely than older participants to report table and cooking salt use, however there was no association between age group and placing a salt shaker on the table. With regards to socioeconomic background, there was no association between SES and table salt use, however those of high SES were more likely to report salt use during cooking and the converse was observed for placing a salt shaker on the table (Table 9).

**Table 7:** Are you trying to cut down on the amount of salt you eat? ^a b^

Group	Sex (*n* = 2268)			Age group (years) (*n* = 2268)						SES (*n* = 2255)			
	Male	Female	*P*-value	18–24	25–34	35–44	45–54	55–65	*P*-value	Low	Mid	High	*P*-value
	%	%		%	%	%	%	%		%	%	%	
Yes	58.2	57.2	0.653	35.6	41.2	37.5	42.4	50.1	**<0.001**	41.8	40.7	43.9	0.416
No	41.8	42.8		64.4	58.8	62.5	57.6	49.9		58.2	59.3	56.1	

^a^Association between categorical variables assessed by Chi-square test. Significant findings (i.e. *P* < 0.05) are shown in bold

^b^Participants who responded ‘don’t know’ *n* = 130 were removed from this analysis

**Table 8:** Behavioural practices to reduce salt intake performed in the past month by demographic characteristics^a^

Group	Sex (*n* = 2398)			Age group (years) (*n* = 2398)						SES (*n* = 2377)			
	Male	Female	*P*-value	18–24	25–34	35–44	45–54	55–65	*P*-value	Low	Mid	High	*P*-value
	%	%		%	%	%	%	%		%	%	%	
Used spices/herbs instead of salt when cooking													
Often or always do this	45.4	59.3	**<0.001**	53.0	47.1	48.8	55.1	60.9	**<0.001**	47.5	52.0	58.4	**<0.001**
Sometimes do this	29.1	22.5		27.5	29.7	26.2	23.3	21.9		26.1	27.1	23.5	
Never or rarely do this	21.0	15.9		14.3	19.9	20.5	19.5	15.0		22.6	16.7	16.1	
Does not apply to me	4.5	2.3		5.2	3.3	4.5	2.1	2.2		3.8	4.2	2.0	
Avoided eating food from fast food restaurants (e.g. McDonalds, KFC, Pizza Hut)													
Often or always do this	47.1	57.1	**<0.001**	44.2	47.1	47.3	51.5	67.2	**<0.001**	41.0	48.6	64.0	**<0.001**
Sometimes do this	24.0	24.0		24.7	27.5	27.5	23.2	18.2		25.8	28.3	19.6	
Never or rarely do this	25.3	17.1		26.3	22.7	22.4	22.0	14.1		30.1	20.7	14.4	
Does not apply to me	3.5	1.8		4.8	2.7	2.8	3.3	0.5		3.1	2.4	2.0	
Avoided eating packaged, ready-to-eat foods													
Often or always do this	41.5	52.2	**<0.001**	41.8	41.8	43.5	46.9	59.1	**<0.001**	37.2	46.1	56.0	**<0.001**
Sometimes do this	27.8	27.6		27.5	30.3	29.6	27.6	23.9		27.9	28.9	26.4	
Never or rarely do this	27.5	18.6		26.3	25.4	24.1	23.2	16.2		32.3	22.2	16.2	
Does not apply to me	3.2	1.6		4.4	2.5	2.8	2.3	0.8		2.6	2.8	1.4	
Purchased foods labelled “no added salt”, “salt reduced” or “reduced sodium”													
Often or always do this	32.6	40.8	**<0.001**	29.4	34.2	32.4	38.3	46.3	**<0.001**	32.2	35.8	42.0	**0.001**
Sometimes do this	32.7	29.9		32.7	33.6	34.5	30.9	25.4		31.7	32.3	29.6	
Never or rarely do this	31.3	27.8		33.9	30.1	30.6	28.6	26.3		33.3	28.9	27.0	
Does not apply to me	3.4	1.5		4.0	2.1	2.5	2.2	2.0		2.8	3.0	1.4	
Avoided eating food from an Asian style restaurant or takeaway store (e.g. Chinese, Thai, Indian)													
Often or always do this	32.4	34.6	**<0.001**	27.1	31.8	31.5	31.7	41.4	**0.001**	28.4	31.7	38.6	**<0.001**
Sometimes do this	26.1	32.4		31.1	30.9	27.5	30.7	29.0		27.6	31.3	29.8	
Never or rarely do this	37.3	29.4		36.2	33.0	36.2	33.5	27.8		38.7	32.9	29.1	
Does not apply to me	4.2	3.6		5.6	4.3	4.8	4.1	1.8		5.3	4.1	2.5	
Looked at a food label to check the salt/sodium content of a food item													
Often or always do this	28.3	32.0	**0.014**	23.5	27.9	29.4	29.4	38.5	**<0.001**	25.5	30.2	34.1	**0.001**
Sometimes do this	26.3	25.6		28.3	24.5	25.5	25.5	24.6		25.5	28.4	24.2	
Never or rarely do this	42.3	40.9		43.8	44.7	43.2	43.2	35.7		46.5	38.7	40.3	
Does not apply to me	3.1	1.5		4.4	2.9	1.9	1.9	1.2		2.5	2.7	1.4	
When eating out, asked to have your meal prepared without salt													
Often or always do this	16.3	8.6	**<0.001**	11.9	17.8	15.2	6.8	8.6	**<0.001**	10.3	9.9	14.5	**<0.001**
Sometimes do this	14.3	9.1		10.4	12.7	13.5	11.5	8.7		9.4	14.5	10.2	
Never or rarely do this	65.8	79.6		72.5	65.4	68.5	79.4	80.5		77.0	72.2	72.9	
Does not apply to me	3.6	2.7		5.2	4.1	2.8	2.3	2.2		3.3	3.4	2.4	

^a^Association between categorical variables assessed by Chi-square test. Significant findings (i.e. *P* < 0.05) are shown in bold

**Table 9:** Discretionary salt use behaviours by demographic characteristics^a,b^

Group	Sex			Age group (years)						SES			
	Male	Female	*P*-value	18–24	25–34	35–44	45–54	55–65	*P*-value	Low	Mid	High	*P*-value
	%	%		%	%	%	%	%		%	%	%	
How often do you add salt to your food at the table? (sex & age group *n* = 2381, SES *n* = 2364)													
Often or always	28.6	20.8	**<0.001**	23.3	29.2	27.5	23.1	18.4	**<0.001**	25.4	24.5	23.0	0.668
Sometimes	25.4	23.1		27.3	25.4	25.3	22.4	22.1		25.0	23.7	23.8	
Never or rarely	46.0	56.1		49.4	45.4	47.2	54.5	59.5		49.6	51.8	53.2	
In the food you eat at home, how often is salt added during cooking? (sex & age group *n* = 2369, SES *n* = 2354)													
Often or always	42.3	33.1	**<0.001**	48.6	49.3	39.1	31.4	25.0	**<0.001**	41.0	32.9	43.2	**<0.001**
Sometimes	27.4	24.1		26.1	26.2	28.5	25.5	22.2		27.1	25.8	24.2	
Never or rarely	30.3	42.8		25.3	24.5	32.4	43.1	52.8		31.9	41.3	32.6	
Do you place a salt shaker on your table at meal times? (sex & age group *n* = 2378, SES *n* = 2363)													
Often or always	32.4	24.7	**<0.001**	29.5	30.2	27.7	26.7	27.1	0.927	35.4	28.2	23.1	**<0.001**
Sometimes	20.6	19.2		20.1	20.1	20.0	20.2	18.9		17.9	21.2	20.1	
Never or rarely	47.0	56.1		50.4	49.7	52.3	53.1	54.0		46.7	50.6	56.8	

^a^Association between categorical variables assessed by Chi-square test. Significant findings (i.e. *P* < 0.05) are shown in bold

^b^Participants who responded ‘don’t know’ were removed from this analysis

## Discussion

Our findings demonstrate that while certain aspects of salt related knowledge are well understood among Victorian adults, a number of gaps exist which vary across socio-demographic characteristics that could be targeted in consumer awareness campaigns. Consistent with past studies [33] most participants were aware of the harmful effect of excess salt on health and the link with particular conditions such as raised blood pressure and coronary heart disease. Fewer were aware of the link with other health conditions, notably about a third did not identify stroke as a related risk. It appears that the message of high blood pressure and heart disease as a determinant of a high salt intake is reaching the public but there is scope to raise greater awareness for stroke risk, particularly given stroke is the third leading cause of death in Australia [34] and the evidence linking high salt intake to stroke is well established [35].

Majority of participants were aware that most Australians eat too much salt, yet very few believed their own individual salt intake would exceed dietary recommendations. The finding, whereby consumers underestimate their own intake is consistently reported across other population groups [28, 29, 36, 37]. Consumers may misjudge their own intake for at least two reasons. Firstly, knowledge of dietary salt recommendations was poor. Secondly, although participants were aware that most salt comes from processed foods, a more thorough understanding may be lacking of how widespread salt is across the food supply and that everyday food items, such as bread and cereal products, can provide substantial amounts of salt to the diet. . There is a clear need to raise awareness of the current high salt intakes seen across the community as well as what foods contribute salt to the diet. In particular, efforts should be focused in reaching those sub-groups of the population which generally displayed less knowledge of these factors (i.e. males, younger to middle age adults (i.e. 25–44 years) and those of lower socioeconomic background.

Overall only 41% of participants believed that their health would improve if they reduced the amount of salt in their diet. Similarly, less than half were concerned about the amount of salt in their diet. These findings are not surprising, given the majority did not view their own intake as being high. Previous studies have shown that adults who believe salt reduction is important or who are concerned with the amount of salt in their diet are more likely to be taking action to reduce dietary salt [29] or engaging in salt related behaviours such as checking the sodium information on food labels and purchasing foods labelled salt reduced [26, 37]. Hence, shifting Victorians’ attitudes related to concern for dietary salt may be an important precursor for salt related behaviour change. Particularly, in those sub-groups where concern for salt in the diet was the lowest (e.g. males, younger participants (e.g. 18–24 years) and those of lower socioeconomic background. Just under half of survey participants reported difficulty understanding the sodium content on food labels and less than one third of participants regularly use food labels to check the salt content of foods, suggesting the sodium information on food labels is inadequate. In Australia, nutrition labels are required to display the sodium content of food per serve and per 100 g within the nutrition information panel (NIP) and the salt content is not provided [38]. This requires consumers to perform a calculation to determine the salt content of foods. However, only 33% of participants knew there was a difference between salt and sodium indicating it would be challenging for consumers to interpret how the sodium content of a food product relates to their overall salt intake. In the United Kingdom (UK) some food manufacturers opt to voluntarily provide information on the salt content (g) of a food product on the food label [39]. A similar approach in Australia could help consumers find lower salt foods, particularly in view of consumer awareness campaigns that target ‘salt’ rather than ‘sodium’. The survey also found that only one quarter of participants could correctly identify there were no health differences between regular salt and gourmet salts, suggesting that accurate information to inform food choices is not reaching consumers. These findings identify key issues to address in a public awareness campaign to enable individuals to understand and use food labels correctly and better inform healthy choices.

In Australia, the amount spent on fast food and dining out represents the highest component of household expenditure on food [40]. Participants reported a limited availability of low-sodium meal options when dining out, indicating that the restaurant and food service industry remain an important inclusion for future salt awareness and reduction programs. There is some evidence to indicate that the average sodium content of fast foods available from leading Australian chains fell, slightly, during the period 2009–2012 [41]. However, of note the average sodium content per serving remained unchanged during this period and at high levels (605 mg/serving) [41]. l.

Consumers attributed collective responsibility for reducing the amount of salt consumed by the Australian population, between individuals (89%) and the food industry (including food manufacturers (81%), fast food chains (77%) and chefs (76%). This study suggests there is strong consumer support for greater legislative and policy action on salt reduction in Australia.

Legislation which limits the amount of salt permitted (i.e. salt content targets) in processed foods has been identified as the most cost-effective strategy in the primary prevention of cardiovascular disease and lead to immediate and significant improvements in population health outcomes [42, 43]. hile voluntary salt content targets are a cost-effective approach, the introduction of mandatory salt content targets on processed foods would provide greater health benefits, averting 18% of Disability Adjusted Life Years (DALY) which are attributable to excess salt consumption in Australia [43]. This is 20 times greater than the proportion of DALYs (0.88%) which would be averted through the use of voluntary salt content targets [43]. [43]. If regulatory salt content limits were implemented, in addition to antihypertensive therapy, this would lead to a significant reduction in lifetime health expenditure saving $4.2 billion in Australia [42]. It is acknowledged that the addition of some salt to manufactured foods is required for functional purposes, including the control of microbial growth, gluten formation in bread and starter culture activity in cheese [44]. However the large variation in salt content within these products [45–47] indicates the feasibility to reduce salt. The Australian Federal Government previously established voluntary sodium targets for 9 food categories under the Food and Health Dialogue which operated 2009–2013 [48]. While there is renewed opportunity for a greater reduction in sodium content of foods with the establishment of the Healthy Food Partnership in 2015, there are no indications that legislative changes to mandate sodium content are being considered by the federal government. Progressive targets and robust monitoring are required to increase the effectiveness of voluntary approach to salt reduction in the current political environment. A further opportunity to incentivise reformulation among food manufacturers is the use of marketing restrictions which are dependent on nutrient profiling. For example the National Heart Foundation of New Zealand’s “Pick the Tick” food label programme encouraged food manufacturers to reduce the amount of salt in breakfast cereals, breads and margarines, resulting in the removal of 33 t of salt from the food supply during 1998–1999, as food manufacturers reduced the amount of sodium in breakfast cereals, breads and margarines to qualify for use of the “Pick the Tick” logo on food products [49].

The reported use of discretionary salt in this sample of Victorian adults is higher compared to national estimates. In the 2011–12 Australian Health Survey 30% of adults reported adding salt during cooking ‘very often’ and 14% reported adding salt at the table ‘very often’ [50]. Comparatively, in the present study 38% reported adding salt during cooking ‘always/often’ and 25% reported adding salt at the table ‘always/often’. These differences may in part be explained by different response options between surveys and the inclusion of a wider age range of participants in the AHS (e.g. 19 years +). Importantly, it has been shown in Victorian adults that salt intake was 0.7 g/d higher in those who reported adding salt at the table and when cooking, compared to those who reported never or rarely adding salt [51]. Together, these findings indicate the need for education messages targeting discretionary salt practices among Victorian adults. Furthermore, our finding that discretionary salt use behaviours differed by socio-demographic characteristics (i.e. males and younger participants more likely to use table and cooking salt), indicate that such messages should be tailored for specific sub-groups of the population. In the UK, self-reported table salt use, which is subject to the inherent limitation of social desirability bias, among adults significantly declined following the implementation of the 2004 Food Standards Agency salt reduction campaign [51]. This was a comprehensive campaign that targeted consumer awareness of high salt intakes and food sources of salt as well as encouraged the food industry to reformulate food products to contain less salt. Importantly, findings from the UK demonstrate the potential for public awareness campaigns to shift discretionary salt use behaviours within the population. With regards to lowering population salt intake it is acknowledged that a combination of strategies is required, which includes product reformulation of lower sodium foods combined with strategies e.g. marketing and consumer education, that seeks to improve overall diet quality.

A strength of the current study includes the large sample size combined with the use of three recruitment methods to capture participants of varying socio-demographic background. However a limitation of the study is that compared to the Victorian population the sample was slightly over-represented of females and older participants, hence limiting the generalizability of the findings to the general population. Furthermore, the low response rate (19% via consumer research panel and 14% via online shopping centre) may introduce non-response bias, as survey responders may be more interested in diet and health. In addition the questionnaire was not validated, however the questions were modelled on those used in previous surveys [22–31] and pilot testing was conducted to improve readability and participant comprehension. Finally, the survey was based on self-reported data, which may be different from actual behaviour.

## Conclusion

This study provides a preliminary assessment of the relevant knowledge, attitudes and behaviours related to salt consumption among adults in the state of Victoria, Australia. Public concern about salt is low as many people remain unaware of their own salt intake. There are difficulties interpreting the sodium content of foods and identifying low salt meal options when dining out. A greater individual awareness of the excessive amount of salt consumed and increased availability of lower salt foods is likely to assist in reducing population salt intake. The study highlights the need to provide easy to understand information on the salt content of foods and lower salt levels in processed foods and food sold outside the home to enable consumers to reduce salt intake. There is consumer support for a public awareness campaign and regulatory approaches to reduce the amount of salt in the food supply. Multisector collaboration between the food industry, health agencies and government is required to improve public awareness and to reduce the salt content of foods to decrease population salt consumption in Australia.

## Additional files

Additional file 1: Knowledge, attitudes and behaviours related to dietary salt intake questionnaire. (PDF 162 kb)

Additional file 2: Table S1. Demographic characteristics (unweighted) of participants by sampling method. (PDF 248 kb)

## Abbreviations

KABs: Knowledge, attitudes and behaviours
SEIFA: Socio-Economic Indexes for Areas
SES: Socioeconomic status
BMI: Body mass index
