# Supplementary material for: Knowledge, attitudes and behaviours related to dietary salt among adults in the state of Victoria, Australia 2015
Source: BMC Public Health. 2017 May 30;17:532. doi: 10.1186/s12889-017-4451-0 (PMC5450045; doi:10.1186/s12889-017-4451-0)
Supplement: Supplementary file 1 — Knowledge, attitudes and behaviours related to dietary salt intake questionnaire. (PDF 162 kb) [file 12889_2017_4451_MOESM1_ESM.pdf]

Victorian Survey – Knowledge, Attitudes and Behaviours related to Dietary Salt Intake

Thank you for taking the time to complete this survey. There are two sections to this survey. Please answer each question by selecting the most suitable option.

All responses on this survey will remain anonymous.

SECTION ONE – DEMOGRAPHIC INFORMATION

In this section we are interested in finding out a little bit of background information about you. This information will help us compare the answers of respondents from different backgrounds.

Q1. What is your sex? *Source: [1]*

Male
Female

Q2. What is your age in complete years? *Source: [1]*

____

Q3. In which country were you born? *Source: [2]*

Australia (includes External Territories)
United Kingdom (incl. England, Scotland, Wales, Northern Ireland)
New Zealand
Italy
Greece
China
Vietnam
Lebanon
Other, please specify _______________________
Don’t know
Prefer not to answer

Q4. Do you speak a language other than English at home?
(If more than one language, indicate the one that is spoken most often) *Source: [2]*

No, English only
Yes, Italian
Yes, Greek
Yes, Cantonese
Yes, Mandarin

Yes, Arabic
Yes, Vietnamese
Yes, German
Yes, Spanish
Yes, Tagalog (Filipino)
Yes, Other (please specify) ____________________
Don't know
Prefer not to answer

Q5. What is the postcode of your residential address?

____

Q6. What is the highest level of education and training you have completed? *Source: [2]*

Never attended school
Some primary school
Completed primary school
Some high school (i.e Year 7 to Year 11, Form 1 to Form 5)
Completed high school (i.e. Year 12, Form 6, HSC)
TAFE or Trade Certificate or Diploma but did not complete Year 12 at secondary school
TAFE or Trade Certificate or Diploma and also completed Year 12 at secondary school.
University, or some other Tertiary Institute degree, including post university (i.e. postgraduate diploma, Master's degree, PhD)
Other, please specify _______________________
Don't know
Prefer not to answer

Q7. Have you ever been diagnosed with or suffered from one or more of the following conditions? *(You may select more than one)*

Heart disease
Stroke
High blood pressure
Heart attack

Other (please specify: ___________)
Can't recall/ Don't know
No

Q7.1 (if Yes to High blood pressure response)

Do you currently take medication for the control of your blood pressure? *Source: [3]*

Yes
No

Q8. Have you ever received any advice from your doctor or a health professional to reduce your intake of salt/sodium and/or salty foods? *Source: [3]*

Yes
No
Cant' recall

Q9. How tall are you without shoes?

You can enter this in cm or feet and inches. **Only fill in one option.** *Source: [1]*

_____________ Centimetres (e.g. 165)

OR

_____________ Feet and inches (e.g. 5 feet 4 inches)

Q10. How much do you weigh without clothes and shoes?

You can enter this in kilograms OR pounds and stones. **Only fill in one option.** *Source: [1]*

______________ Kilograms (e.g. 74)

OR

______________Stones and pounds (e.g. 11st 10lbs)

Q11. Are you the main person who does the grocery shopping in your household? *Source [4]*

Yes
No
I share the responsibility

SECTION TWO – YOUR VIEWS ON SALT INTAKE

[Questions related to knowledge]

In this section we are interested in finding out more about your views on dietary salt.

Q12. On Australian food products information about the amount of sodium within a food product is displayed on the food label. What is the relationship between salt and sodium? *Source: Modified [5, 4, 6]*

They are exactly the same
Salt contains sodium
Sodium contains salt
Don't know

Q13. In general, how much salt do you think Australians eat? *Source: Modelled of [7, 8]*

Far too much
Too much
Just the right amount
Too little
Far too little
Don't know

Q14. Which of the following do you think is the main source of salt in the Australian diet? *Source: Modelled of [5, 7]*

Salt added during cooking or at the table
Salt from processed foods such as breads, sausages and cheese
Salt from natural food sources
Don't know

Q15. Health professionals recommend that we should eat no more than a certain amount of salt **each day**. How much salt do you think this is? *Source: Modelled of [4, 6, 9]*

3 grams (about ½ a teaspoon)
5 grams (about 1 teaspoon)
8 grams (about 1 and a ½ teaspoons)
10 grams (about 2 teaspoons)
15 grams (about 3 teaspoons)
Don't know

Q16. How do you think your daily salt intake compares to the amount of salt recommended by health professionals? *Source: Modified [5, 4]* [Note Q16 attitudes questions]

I eat less salt than recommended
I eat about the right amount of salt
I eat more salt than recommended
I don't know

Q17. Do you think that eating too much salt could damage your health? *Source: [10, 11]*

Yes
No
Don't know

Q18. Which, if any, of the following conditions do you think is linked to eating too much salt?

Please make sure you select an option for each line. *Source: Modelled of [5, 10, 4, 8]*

**Health problem**	**Yes**	**No**	**Don't know/Not sure**
High bood pressure			
Kidney disease			
Heart disease/heart attack			
Stroke			
Stomach cancer			

[Questions related to attitudes]

Q19. Please indicate on the scale below how concerned you are about each of the following food related issues?

Please make sure you select an option for each line. *Source: Modelled of [12, 13]*

	Not at all concerned	Not very concerned	Somewhat concerned	Very concerned	Extremely concerned
Healthy eating					
The amount of sugar in food					
The amount of salt in food					
The amount of fat in food					
The amount of saturated fat in food					
The amount of kilojoules/calories in food					

Q20. Please indicate on the scale below how much you agree or disagree with the following statements.

Please make sure you select an option for each line. *Source: Modified [12, 4]*

**Statement**	**Strongly disagree**	**Disagree**	**Neither agree nor disagree**	**Agree**	**Strongly agree**
Himalayan salt, pink salt, sea salt and gourmet salts are healthier than regular table salt					
I believe salt needs to be added to food to make it tasty					
My health would improve if I reduced the amount of salt in my diet					
It is hard to understand sodium information displayed on food labels					
When eating out at restaurants/cafes/pubs, I find that lower salt options are not readily available or only in limited variety					
There should be laws which limit the amount of salt added to manufactured foods					

Q21. From the list below please rate how responsible you think each group is for reducing the amount of salt Australians eat.

Please make sure you select an option for each line. *Source: Modified [14]*

**Group**	**Not at all responsible**	**Somewhat responsible**	**Responsible**	**Very responsible**	**Don't know**
Government					
Food manufacturers					
Business (e.g. supermarkets, local markets)					
Chefs preparing foods in restuarants/pubs/cafes					
Friends/family					
Yourself					
Fast food chains					

[Questions related to behaviours]

Q22. How often do you add salt to your food at the table? *Source: [8]*

Always
Often
Sometimes
Rarely
Never
Don't know

Q23. In the food you eat at home, how often is salt added during cooking? *Source: [8]*

Always
Often
Sometimes
Rarely
Never
Don't know

Q24. Do you place a salt shaker on your table at meal times? *Source: Modified [15]*

Always
Often
Sometimes
Rarely
Never
Don't know

Q25. Are you trying to cut down on the amount of salt you eat? *Source: Modified [13, 6, 11, 8]*

Yes
No
Don't know

Q26. Below are some common ways to lower the amount of salt in your diet. In the past month, to what extent have you personally done any of the following?

Please make sure you select an option for each line. *Source: Modelled from [12, 8]*

	Never do this	Rarely do this	Sometimes do this	Often do this	Always do this	Does not apply to me
Looked at a food label to check the salt/sodium content of a food item						
Avoided eating packaged, ready-to-eat foods						
Used spices/herbs instead of salt when cooking						
Avoided eating food from fast food restaurants (e.g. McDonalds, KFC, Pizza Hut)						
Avoided eating food from an Asian style restaurant or takeaway store (e.g. Chinese, Thai, Indian)						
Purchased foods labelled “no added salt”, “salt reduced” or “reduced sodium”						
When eating out, asked to have your meal prepared without salt						

Q27. On some food products a health star rating is displayed on the food label.

[insert image of health star rating label]

Do you use the health star rating information to choose any of the following products?

	Usually	Sometimes	Never	Don't know
Breakfast cereals				
Cheese				
Bread				

Q28. Are you aware of VicHealth's initiative to reduce salt intake within the Victorian population?

Yes
No
Don't know

Q29. Are you a parent or caregiver for a child or children under the age of 18 years?

No
Yes, I'm a parent
Yes, I care for a child/children

(if no directed to end of survey)

You have completed the Survey! Please hit the submit button.

Thank you for your time.

References

1. Australian Institute of Health and Welfare. National Health Data Dictionary version 16. Canberra2012.
2. VicHealth. VicHealth Indicators Survey 2011. 2012. https://www.vichealth.vic.gov.au/programs-and-projects/vichealth-indicators-survey-2011. Accessed 23 Sep 2016.
3. Booth A. Implementation strategies and effectiveness of nutritional messages for improving health. Victoria: Deakin University; 2007.
4. Grimes CA, Riddell LJ, Nowson CA. Consumer knowledge and attitudes to salt intake and labelled salt information. Appetite. 2009;53(2):189-94.
5. Australian Division of World Action on Salt and Health. 2007 Survey of Australian Consumer Awareness and Practices Relating to Salt Report. 2007. http://www.awash.org.au/wp-content/uploads/2012/10/AWASH_ConsumerSurveyReport_2007_05_15.pdf. Accessed 26th May 2015.
6. New Zealand Food Safety Authority. Salt Consumer Survey. 2011. http://www.foodsafety.govt.nz/elibrary/industry/salt-survey.pdf. Accessed 26th May 2015.
7. Papadakis S, Pipe AL, Moroz IA, Reid RD, Blanchard CM, Cote DF et al. Knowledge, attitudes and behaviours related to dietary sodium among 35- to 50-year-old Ontario residents. Can J Cardiol. 2010;26(5):e164-9.
8. World Health Organization. WHO STEPS Instrument (Core and Expanded) v3.1. 2015. http://www.who.int/chp/steps/instrument/en/. Accessed 26th May 2015.
9. Sarmugam R, Worsley A, Flood V. Development and validation of a salt knowledge questionnaire. Public Health Nutr. 2014;17(5):1061-8.
10. Consensus Action on Salt and Health. Salt and Your Health. TNS Public Opinion Survey. 2009. http://www.actiononsalt.org.uk/Docs/33386.pdf. Accessed 5th Aug 2015.
11. World Health Organization, Pan American Health Organization. Protocol for Population Level Sodium Determination in 24-hr Urine Samples. Canada: WHO/PAHO Regional Expert Group for Cardiovascular Disease Prevention through Population-wide Dietary Salt Reduction2010.
12. Arcand J, Mendoza J, Qi Y, Henson S, Lou W, L'Abbe MR. Results of a National Survey Examining Canadians' Concern, Actions, Barriers, and Support for Dietary Sodium Reduction Interventions. Canadian Journal of Cardiology. 2013;29(5):628-31.
13. Food Standards Agency, COI Communications. Consumer Attitudes to Food Standards Wave 5 - 2004. London2005.
14. Newson RS, Elmadfa I, Biro G, Cheng Y, Prakash V, Rust P et al. Barriers for progress in salt reduction in the general population. An international study. Appetite. 2013;71:22-31.
15. Grimes CA, Baxter JR, Campbell KJ, Riddell LJ, Rigo M, Liem DG et al. Cross-Sectional Study of 24-Hour Urinary Electrolyte Excretion and Associated Health Outcomes in a Convenience Sample of Australian Primary Schoolchildren: The Salt and Other Nutrients in Children (SONIC) Study Protocol. JMIR Res Protoc. 2015;4(1):e7.
