# Supplementary material for: Knowledge, attitudes and behaviours related to dietary salt among adults in the state of Victoria, Australia 2015
Source: BMC Public Health. 2017 May 30;17:532. doi: 10.1186/s12889-017-4451-0 (PMC5450045; doi:10.1186/s12889-017-4451-0)
Supplement: Supplementary file 2 — Demographic characteristics (unweighted) of participants by sampling method. (PDF 248 kb) [file 12889_2017_4451_MOESM2_ESM.pdf]

Additional file 2. Supplementary table 1. Demographic characteristics (unweighted) of participants by sampling method

**Characteristic**	**Total Sample**		**Shopping Centres**		**Facebook**		**Consumer Research Panel**		**P-value** [1]
	**n**	**%**	**n**	%	n	%	n	%	
**Total participants**	**2398**		**373**		404		1621		
**Gender**									
Male	1046	43.6	112	30.0	65	16.1	869	53.6	<0.001
Female	1352	56.4	261	70.0	339	83.9	752	46.4	
**Age (years) mean and SD**	42.7	13.4	43.2	13.7	48.5	13.9	41.2	12.8	0.05
**Age (years)**									
18-24 y	251	10.5	41	11.0	49	12.1	161	9.9	<0.001
25-34 y	512	21.3	75	20.1	31	7.8	406	25.1	
35-44 y	527	22.0	80	21.5	37	9.2	410	25.3	
45-54 y	514	21.4	70	18.8	97	24.1	347	21.4	
55-65 y	594	24.8	107	28.7	190	47.0	297	18.3	
**Country of Birth**									
Australia	1915	79.9	264	70.8	332	82.2	1319	81.4	<0.001
United Kingdom	86	3.5	12	3.2	22	5.5	52	3.2	
New Zealand	29	1.2	4	1.1	7	1.7	18	1.1	
Italy	10	0.4	0	0.0	1	0.3	9	0.6	
Greece	11	0.5	1	0.3	2	0.5	8	0.5	
China	30	1.2	10	2.7	1	0.3	19	1.2	
Vietnam	14	0.6	2	0.5	1	0.3	11	0.7	
Lebanon	4	0.2	1	0.3	1	0.3	2	0.1	
Other	271	11.3	77	20.6	35	8.7	159	9.8	
Prefer not to answer or don't know	28	1.2	2	0.5	2	0.5	24	1.5	
**Do you speak a language other than English at home?**									
Yes	409	17.1	104	27.9	45	11.1	260	16.0	<0.001
No, English only	1969	82.1	267	71.6	357	88.4	1345	83.0	

Prefer not to answer or don’t know	20	0.8	2	0.5	2	0.5	16	1.0	
**Socioeconomic status based on highest level of education**[2]									
High SES	1020	42.9	188	50.8	191	47.3	641	40.0	<0.001
Mid SES	675	28.4	83	22.4	102	25.3	490	30.6	
Low SES	682	28.7	99	26.8	111	27.5	472	29.4	
**Height (cm) mean and SD**	169.3	10.2	167.2	10.0	166.9	8.6	170.4	10.4	<0.001
**Weight (Kg) mean and SD**	77.6	18.7	76.26	18.1	77.07	17.7	78.14	19.1	0.18
**BMI (kg/m2) mean and SD**	27.0	6.1	27.3	6.1	27.68	6.2	26.8	6.1	0.03
**Weight category**									
Underweight (BMI <18.5)	68	3.2	10	2.8	9	2.4	49	3.5	0.09
Healthy weight (BMI =>18.5 & <25.0)	846	39.6	146	40.3	131	34.3	569	40.9	
Overweight (BMI =>25.0 & <30.0)	690	32.3	105	29.0	141	36.9	444	31.9	
Obese (BMI =>30.0)	532	24.9	101	27.9	101	26.4	330	23.7	
**Diagnosed with a chronic condition**									
Yes	705	29.4	103	27.6	135	33.4	467	28.8	0.17
No	1659	69.2	264	70.8	267	66.1	1128	69.6	
Don't know/can't recall	34	1.4	6	1.6	2	0.5	26	1.6	
**Are you the main person who does the grocery shopping in your household?**									
Yes	1654	69.0	241	64.6	266	65.8	1147	70.8	0.04
No	239	10.0	49	13.1	39	9.7	151	9.3	
No, I share the responsibility	505	21.0	83	22.3	99	24.5	323	19.9	

[1] Chi-squared test for categorical data and one-way ANOVA for continuous data to assess differences between recruitment methods.

[2] n=2,377 as participants who responded “don’t know” or “prefer not to answer” n=21 were excluded.
